# The enigma of the B chromosome-specific behaviour

**DOI:** 10.3389/fpls.2026.1888257

**Published:** 2026-07-16

**Authors:** Lucie Hloušková, Miroslava Karafiátová, Jan Bartoš

**Affiliations:** 1 Centre of Plant Structural and Functional Genomics, Institute of Experimental Botany of the Czech Academy of Sciences, Olomouc, Czechia; 2 Department of Cell Biology and Genetics, Palacký University, Olomouc, Czechia

**Keywords:** B chromosome, centromere, chromosome elimination, chromosome segregation, nondisjunction, supernumerary chromosomes

## Abstract

Supernumerary (B) chromosomes are widespread genomic elements that persist through non-Mendelian inheritance by exploiting host cellular mechanisms, yet the basis of their selective transmission and elimination remains poorly understood. This review integrates current knowledge on chromosome-specific behaviour, with particular emphasis on the centromere function, kinetochore assembly, epigenetic chromatin states, non-coding RNAs, and sequence composition. We examine how perturbations in these systems can skew chromosome segregation, or lead to chromosome elimination, and consider growing evidence that B chromosomes may themselves encode or modulate factors influencing these processes. Their repeat-rich architecture and enrichment in chromosome-specific satellite DNA are also discussed as contributors to their recognition by the cellular machinery. By unifying structural, epigenetic, and genetic perspectives, this review outlines a framework for understanding chromosome drive and elimination and highlights key directions for future research.

## 1 Introduction

Generally, chromosomes behave uniformly during division to ensure faithful transmission of genetic material. Uncorrected deviations can lead to aneuploidy, impaired cell function, or reduced fitness of the organism (reviewed by Santaguida and Amon, 2015). However, this is not the case for some chromosomes, where chromosome-specific behaviour is a conditional survival strategy. Specifically, this concerns supernumerary chromosomes.

**Figure 1:**
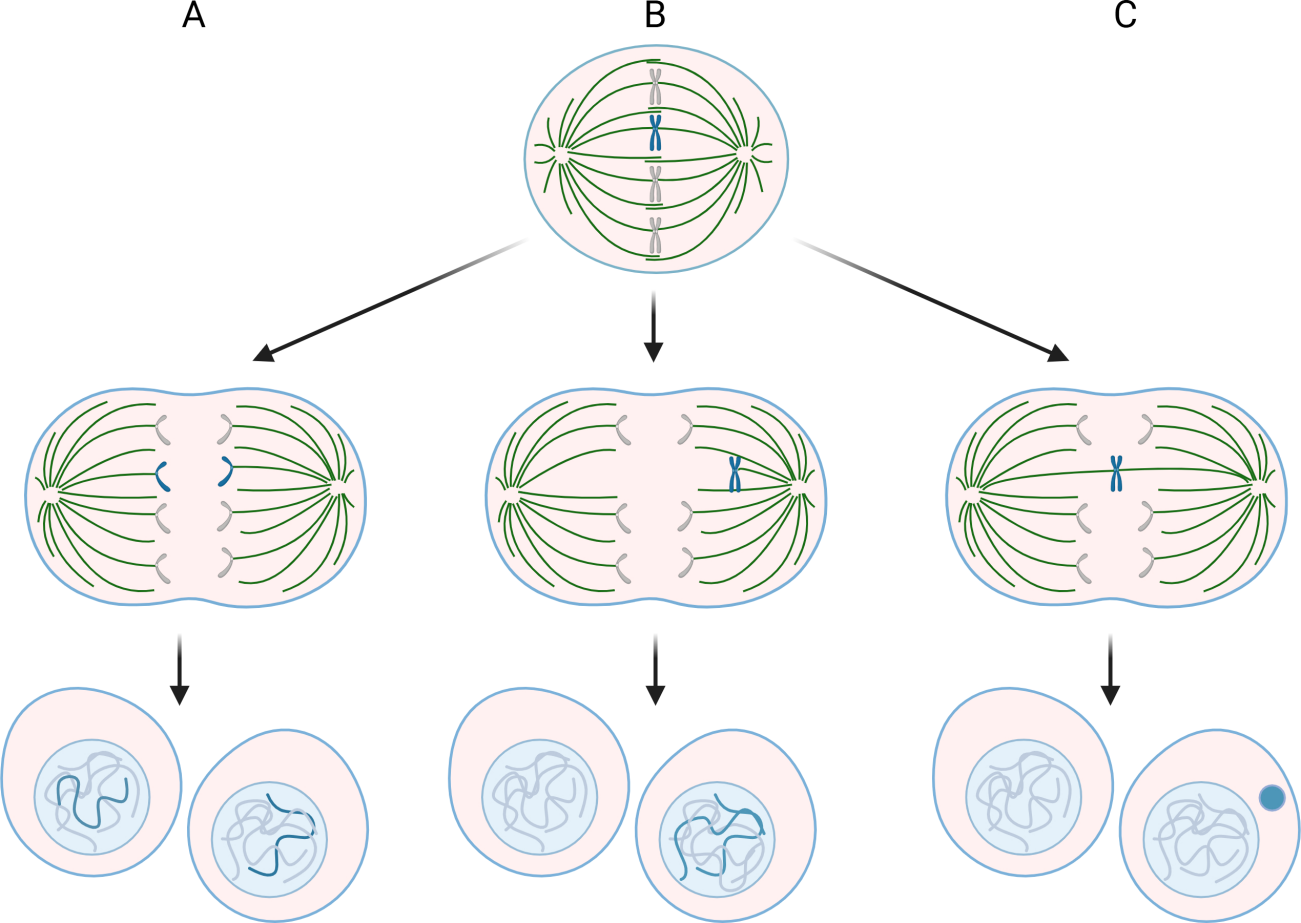
Possible scenarios of B chromosome (in blue) behaviour during cell division. Schematic illustration of standard B chromosome transmission **(A)**, B chromosome nondisjunction **(B)**, B chromosome elimination **(C)**. In most of the divisions, B chromosome segregates regularly **(A)**. During critical division it shows a modified performance. During anaphase, the B chromosome can undergo nondisjunction and be preferentially segregated to one daughter cell **(B)**, or it can lag behind other chromosome and subsequently be eliminated in the form of micronucleus **(C)**. Created in BioRender. Hloušková, L. (2026) https://BioRender.com/lx3yoo9.

Supernumerary chromosomes, also called B chromosomes, or PSR (paternal sex ratio) chromosomes in certain insects (Van Vugt et al., 2009; Aldrich et al., 2017), represent non-essential genetic elements widespread across kingdoms (D'Ambrosio et al., 2017). B chromosomes are highly variable in their abundance and structure across taxa. Their occurrence ranges from rare individuals to frequencies exceeding 50% of natural populations, while their size can vary from small microchromosomes to elements approaching the size of standard chromosomes (Camacho et al., 2000; Houben, 2017). B chromosomes represent a highly diverse group of genomic elements that differ from standard chromosomes not only in their dispensability but also in their sequence composition, inheritance patterns, and interactions with cellular processes (**Table 1**). Essentially, their biology is characterized by abnormal behaviour (**Figure 1**) generally acting in their favour. As first, they mostly persist in populations by chromosome drive, a set of mechanisms that promote their over-representation in gametes or zygotes beyond Mendelian expectations (reviewed by Camacho et al., 2000; Houben, 2017). Chromosome drive can arise through diverse processes, including but not limited to meiotic or mitotic nondisjunction, asymmetric spindle behaviour, and preferential segregation into functional gametes (**Figure 1B**; Camacho et al., 2000; Houben, 2017). While many well-studied examples of drive involve nondisjunction during meiosis or pollen mitosis in plants, drive may also operate prior to meiosis through mitotic nondisjunction in the germline, leading to an increased number of B chromosomes in cells entering meiosis (Nur, 1969; Parker et al., 1989; Reed and Werren, 1995; Camacho et al., 2000; Houben, 2017; Lee et al., 2023).

**Table 1:** The key distinctions between standard chromosomes and B chromosomes.

Feature	Standard chromosome	B chromosome
Presence in genome	Essential component of the genome	Dispensable supernumerary chromosomes
Inheritance	Mendelian	Usually non-mendelian (drive)
Population frequency	Typically fixed within species	Variable among individuals and populations or even within different tissues of one individual
Fitness effect	Generally required for viability	Often neutral at low numbers, negative effects at high copy numbers
DNA composition	Contains essential genes	Usually enriched in repetitive DNA; genes are frequently fragmented; genes on B chromosome are not essential for the host organism
Evolutionary interests	Individual fitness, survival and reproduction	Self-transmission

Another mechanism specific for B chromosomes is a chromosome elimination, the selective removal of an entire chromosome (**Figure 1C**; Semple, 1976; Ruban et al., 2020; Karafiátová et al., 2024). In particular divisions, B chromosome lags during the anaphase and is excluded from emerging daughter nuclei. Instead, it remains in cytoplasm as micronucleus which is gradually degraded during subsequent development (Ruban et al., 2020; Karafiátová et al., 2024; Bojdová et al., 2026). In contrast to micronuclei generated by random chromosome segregation errors frequently associated with some diseases, these structures are formed intentionally as a part of development. In plants, B chromosome elimination was well documented in the goatgrass *Aegilops speltoides* and the wild sorghum *Sorghum purpureosericeum* (Ruban et al., 2020; Karafiátová et al., 2024). In both species, elimination of the B chromosome was observed during embryo development. Interestingly, while in goatgrass embryo, B chromosome elimination affects only the radicle (Ruban et al., 2020), it is much intense in sorghum preserving the B chromosome only in embryonic plumule (Karafiátová et al., 2024; Bojdová et al., 2026).

Both mentioned processes represent examples of chromosome-specific behaviour. The teasing questions of what mechanisms underlie recognition of the B chromosome during these processes is hanging in the air for decades and has not been satisfactorily elucidated so far. While targeted nondisjunction has been described prevalently for supernumerary chromosomes, the controlled elimination of specific chromosome or DNA region is more widespread and studies outside the B chromosomes may be inspiring (Wang et al., 2017; Chmielewska et al., 2018). An extreme example of programmed elimination is observed in the jewel wasp *Nasonia vitripennis*, where PSR chromosome drives elimination of the whole paternal genome (Dalla Benetta et al., 2020; Lee et al., 2023). Recent work in *Drosophila melanogaster* further demonstrated that B chromosomes can participate in the suppression of female meiotic drive systems, highlighting their role in intragenomic conflict (Bauerly et al., 2014). Apart from B chromosomes, programmed DNA elimination has been documented in representatives of songbirds, insects, copepods, nematodes, and unicellular ciliates as part of their development (Pigozzi and Solari, 2005; Goday and Esteban, 2001; Gerbi, 2022; Sun et al., 2014; Wang et al., 2017; Bracht et al., 2013).

The precise mechanism that underlies chromosome-specific behaviour has not yet been described for any of the species. However, based on the information currently available, it is reasonable to conclude that this behaviour is governed by B-chromosome encoded features acting through multiple regulatory layers. This review aims to integrate the current insights on how chromosome can develop a unique “make-up” setting them apart from other chromosomes and what are the changes that guide it precisely on its journey through the modified cell division.

## 2 Centromere: a key component in chromosome-specific processes

The centromere plays a vital role in the segregation of chromosomes (reviewed by Sundararajan and Straight, 2022; Westhorpe and Straight, 2013; Verdaasdonk and Bloom, 2011). It is the site where the kinetochore assembles to connect the chromosome to the spindle, thereby ensuring accurate transmission (reviewed by Hara and Fukagawa, 2020). While early models often attributed B chromosome drive and elimination to centromere inactivity or failure (Banaei-Moghaddam et al., 2012; Han et al., 2007), emerging evidence suggest that both processes are governed by more complex changes (Ruban et al., 2020; Chen et al., 2024; Bojdová et al., 2026).

Centromere inactivation might be the primary cause of chromosome elimination like in interspecific hybrids of *Hordeum bulbosum* and *H. vulgare* (Bennett et al., 1976; Finch, 1983; Sanei et al., 2011), where the loss of centromeric histone H3 (CENH3) of one parent results in elimination of its chromosomes (Sanei et al., 2011). Similar effects have been observed in *Arabidopsis* hybrids and engineered CENH3 mutants, where altered CENH3 proteins cause uniparental genome elimination (Ravi and Chan, 2010; Maheshwari et al., 2015). Further, the absence of CENP-A (CENH3) loading into specific regions of holocentric chromosomes of parasitic nematode *Ascaris suum* and *Ceanorhabditis elegans* leads to the diminution of these loci (Kang et al., 2016; Gassman et al., 2012). In contrast, chromosome elimination in other systems occurs despite the presence of apparently functional centromeres, as reported in *Festuca x Lolium* hybrids with gradual elimination of *Festuca* chromosomes (Majka et al., 2023) or in sciarid flies representing a model of tissue-specific chromosome elimination (Goday and Esteban, 2001). Here, eliminating chromosomes show CENH3 loading and microtubule attachment.

Theory of inactive centromeres as a cause of the B chromosome nondisjunction was confidently refuted in rye and *Aegilops speltoides*, where the B chromosomes were proved to be attached to the mitotic spindle during nondisjunction at first pollen mitosis (Banaei-Moghaddam et al., 2012; Wu et al., 2019). In rye, studies combining cytogenetics and immunostaining have shown that B chromosome maintains centromere activity but fail to segregate normally, likely due to prolonged cohesion and its characteristic lagging behaviour (Banaei-Moghaddam et al., 2012). However, centromere inactivation cannot be excluded as an alternative explanation for the specific behaviour of B chromosomes in other species.

**Figure 2:**
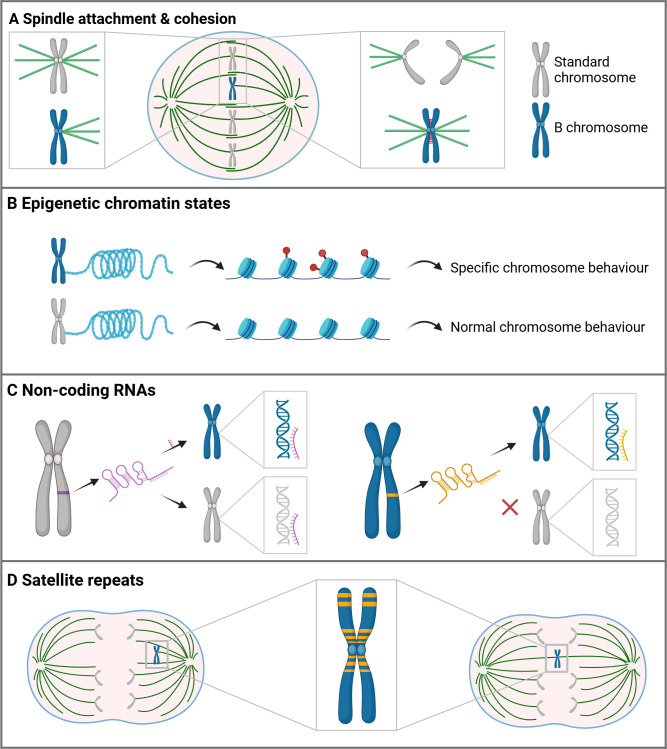
Molecular features considered to be involved in B chromosome-specific behaviour. **(A)** Spindle and cohesin behaviour. Spindle asymmetry (left), together with persistent sister chromatid cohesion (right), can influence chromosome segregation dynamics and promote nondisjunction or lagging behaviour of B chromosome (blue). **(B)** Epigenetic chromatin states. Chromatin organization and chromosome behaviour can be modulated by epigenetic features, including post-translational histone modifications (red marks) such as methylation, acetylation, and phosphorylation, which could lead to specific chromosome behaviour. **(C)** Non-coding RNAs. Non-coding RNAs (ncRNAs) transcribed from standard chromosomes (purple) may interact broadly with multiple chromosomal regions and contribute to general chromatin organization or regulation. In contrast, ncRNAs derived from B chromosome (yellow) may exhibit chromosome-specific binding and preferentially associate with B chromosome sequences. **(D)** Satellite repeats. Accumulation and expansion of repetitive satellite sequences (yellow) within centromeric, pericentromeric or other regions can alter centromere architecture, cohesion properties and spindle interactions, contributing to biased segregation, nondisjunction (left) or chromosome elimination (right). Created in BioRender. Hloušková, L. (2026) https://BioRender.com/mmjz9c8.

Beyond centromere activity itself, spindle asymmetry and persistent chromatid cohesion have also been implicated in chromosome drive across multiple species (**Figure 2A**). While the spindle asymmetry is restricted to specific cell divisions such the first pollen mitosis in plants (reviewed by Gerbi, 2022), the extended chromatid cohesion is a more versatile tool. In rye and *Aegilops speltoides*, spindle asymmetry during the first mitotic division in pollen development has been proposed to influence the B chromosome segregation (Banaei-Moghaddam et al., 2012; Wu et al., 2019). Such asymmetric divisions are a common feature of systems exhibiting nondisjunction associated with B chromosome accumulation (Camacho et al., 2000; Houben, 2017). In addition, in the fly *Sciara* (*Bradysia*), male meiosis I is characterized by a monopolar spindle in which paternal chromosomes are eliminated, showing an example of affected segregation driven by organizational symmetry (Escribá et al., 2011; Greciano and Goday, 2006).

Although it is generally accepted that an essential component of biased segregation lies in the dis-regulation of cohesion release between sister chromatids, there is not much evidence supporting this theory. In maize B chromosome, nondisjunction is facilitated by enhanced cohesion, particularly in pericentromeric regions, which resists spindle pulling forces during division (Han et al., 2007). Likely, altered sister chromatid resolution contribute also to the B chromosome elimination in other systems. Mutations in the cohesion subunits and the cohesion-related genes have been the subject of intense research as it induces chromosomal mis-segregation resulting in tumorigenesis in human (Hill et al., 2016). Defects in cohesion maintenance or release can also result in chromosome instability phenotypes such as aneuploidy, chromosome loss, micronuclei formation, and cohesion fatigue during prolonged metaphase arrest (Daum et al., 2011; Kim et al., 2016; Leylek et al., 2020).

Together, these findings suggest that coordinated alterations in centromere identity and cohesion dynamics underpin B chromosome behaviour, although direct experimental and functional evidence for these interplays is still lacking.

## 3 Epigenetic landscape: histone modifications as determinants of chromosome fate

Post-translational histone modifications (PTMs) are well-known epigenetic tool for regulation in various biological processes (reviewed by Millán-Zambrano et al., 2022; Le et al., 2025). As PTMs can act as a hallmark of retention or elimination (**Figure 2B**), the epigenetic landscape of supernumerary chromosomes has been characterised in the representatives of plants as well as animals (Liu et al., 2025; Cardoso et al., 2019). Namely, the PSR chromosome in *Nasonia vitripennis* manipulates epigenetic regulation on a genome-wide scale. It disrupts the normal patterning of several histone modifications on the paternal chromatin in the sperm when it is present. H3K9me2/3, H3K27me1, and H4K20me1 become abnormally enriched and spread across the paternal set of chromosomes, coinciding with the failure of paternal chromosome condensation during the first mitotic division in embryo and its subsequent elimination. Notably, PSR itself lacks H3K27me1 and H4K20me1 during this stage, which may contribute to its escape from elimination (Aldrich et al., 2017). This strong example shows how a B chromosome can hijack the host’s epigenetic machinery to its own advantage. Comparable associations between histone modification patterns and large-scale chromatin condensation or chromosome silencing have also been reported in other systems, e.g. in mealybugs (Bongiorni et al., 2007; Bain, 2019), ciliates and hagfish (Taverna et al., 2002; Kojima et al., 2010), and grasshoppers (Cabrero et al., 2007). However, despite increasing knowledge of chromatin states on plant B chromosomes (Liu et al., 2025; González-Sanchéz et al., 2014), their functional contribution to chromosome drive or elimination remains poorly understood. Recent epigenetic profiling of the *Drosophila melanogaster* B chromosome revealed enrichment of H3K9me1 and H3K9me2, depletion of H4K5ac and exclusion of RNA polymerase II, indicating that the chromosome forms a transcriptionally repressed chromatin domain (Ferretti et al., 2026). Although these features were not directly linked to chromosome drive, they further support the view that B chromosomes frequently adopt distinctive epigenetic states.

Evidence for role of PTMs in chromosome elimination also comes from germline-restricted chromosomes (GRCs). In the songbird zebra finch, GRCs acquire a densely heterochromatic state during male meiosis, with enrichment of H3K9me2/3, H4K20me2/3, and HP1β, indicative of transcriptional silencing and nuclear compartmentalization (Goday and Pigozzi, 2010). Their chromatin progressively condenses and is eventually eliminated during metaphase II (Pigozzi and Solari, 2005). In Bengalese finch, GRC chromatin shows crosstalk between H3K9me3 and under-phosphorylation of H3S10, possibly due to disrupted Aurora B kinase activity, which again signals its fate for elimination (del Priore and Pigozzi, 2014).

Overall, these examples highlight that chromatin and its epigenetic states might contribute to the B chromosome-specific behaviour. These distinct patterns guide chromosome condensation and can be exploited by selfish chromosomes to bias their transmission. Across taxa, the recurring association between PTMs and programmed elimination has been demonstrated only in isolated examples and additional and broader studies are required to generalize on the role of the epigenetic regulation in the B chromosome specific behaviour.

## 4 Non-coding RNAs: emerging regulators in chromosome-specific processes

Beyond structural and epigenetic factors, growing evidence suggests that noncoding RNAs (ncRNAs), can play significant roles in chromatin regulation and chromosome behaviour across eukaryotes. ncRNAs expressed from B chromosomes, identified recently in multiple species, are now recognized as potential regulatory elements influencing chromosome dynamics, though direct functional evidence remains limited (**Figure 2C**; reviewed by Oliveira et al., 2024; Dalla Benetta et al., 2019). In general, these ncRNAs can modulate chromatin states, influence kinetochore and cohesion dynamics, and potentially act as molecular guides for protein complexes that determine chromosome fate (Moazed, 2009; Borges and Martienssen, 2015).

A pivotal study in rye B chromosomes revealed that a satellite sequence (Non-Coding Repeat, NCR) produces long non-coding RNAs almost exclusively in anthers (Carchilan et al., 2007). These transcripts originate from transcriptionally active heterochromatin, an unusual feature for highly repetitive B-specific DNA. Although the exact role of ncRNA must be further elucidated, there are two main hypotheses describing their effect. Possibly, NCR-derived RNAs help stabilize pericentromeric cohesion of B-sister chromatids during the first pollen mitosis, delaying their separation and facilitating nondisjunction. This hypothesis is supported by the emerging role of ncRNAs in cohesion regulation (reviewed by Kuru-Schors et al., 2021). Mechanistically, such RNAs may act as molecular roadblocks, occupying binding sites of cohesion-release factors or chromatin remodelers at B-specific loci, which is shown as general mechanisms of chromatin-associated ncRNA guiding or sequestering chromatin modifiers (reviewed by Yin and Shen, 2023).

Concerning the elimination and epigenetic patterns discussed in previous sections, small RNAs (21–30 nt) are interesting candidate regulators of chromatin states (Ugarkovic, 2005; Slotkin and Martienssen, 2007). In the PSR chromosome of *Nasonia vitripennis*, transcriptional profiling revealed a subset of small RNAs mapping to PSR-specific satellite repeats, expressed at much higher levels than comparable repeats on A chromosomes (Dalla Benetta et al., 2020). Although the functions of these PSR-associated small RNAs remain unexplored, insights from established RNA-guided chromatin regulatory systems suggests that they may influence chromatin states or recruitment of chromatin-modifying factors (Moazed, 2009). In plants, meiotic small RNAs have been implicated in defining centromere identity, maintaining germline genome stability, and directing heterochromatin assembly (Borges and Martienssen, 2015; Long et al., 2021). This raises the possibility, by analogy to known RNA-mediated chromatin regulation, that B chromosomes or other selfish elements could manipulate silencing pathways in their own favour.

A more speculative but captivating hypothesis is that ncRNAs may contribute directly to the mechanics of chromosome segregation. Emerging work in animals shows that RNA molecules and RNA-binding proteins can influence kinetochore assembly, centrosome function, and microtubule stability (Blower, 2016). Similarly, it is conceivable that B chromosome-derived ncRNAs could modulate spindle-kinetochore dynamics or compete for RNA-binding factors, subtly biasing segregation outcomes, even though no studies have directly tested such mechanisms in B chromosomes yet.

No matter how ingenious the ncRNAs effects are, their role in nondisjunction/elimination is likely not independent of other mechanisms described earlier. Instead, RNA-mediated processes may form a regulatory layer atop the structural and histone modification frameworks, offering sequence-specific guidance and temporal control.

## 5 Sequence composition: a basis for chromosome identity and behaviour

Sequence composition of supernumerary chromosomes is shaped by distinct evolutionary path they take. It provides an important framework for understanding the mechanisms underlying their specific behaviour. Transcriptomic analyses indicate that some B-encoded genes are expressed in a context-dependent manner and may contribute directly to chromosome behaviour. As for elimination, in wild sorghum embryos undergoing B chromosome elimination, several mitosis-related genes, including CENH3, CENP-C, Mis12, Nuf2, and SMC3, are expressed from the B chromosome (Bojdová et al., 2026). These genes are represented by specific variants rather than exact copies of their A chromosomal homologs. Structural modelling of B chromosome-encoded CENH3 and CENP-C revealed amino acid substitutions affecting their predicted interaction interface, raising the possibility that they alter kinetochore assembly or function. The identification of such transcripts is consistent with the idea that B chromosomes can actively modulate kinetochore function and cohesion to manipulate their segregation. Similar gene content has been reported in *Aegilops speltoides* (Boudichevskaia et al., 2020), suggesting that shared mechanistic strategies may operate across species. Apart from the mitotic-related genes, the PSR chromosome of jewel wasp *Nasonia vitripennis*, carries the haploidizer gene required for paternal genome elimination; its perturbation disrupts characteristic histone modification patterns associated with chromosome loss (Dalla Benetta et al., 2020). In parallel, candidate genes linked to drive have also been identified in plants, such as DCR28 and DCR400 in rye, which are associated with microtubule function and sister chromatid cohesion, respectively (Chen et al., 2024). In contrast, maize B chromosome drive appears to rely on multiple cis- and trans-acting elements rather than a single determinant (Ward, 1973; Lamb et al., 2005; Blavet et al., 2021). Together, these findings support the view that B chromosome behaviour is, at least in part, controlled by the B chromosome itself.

The enrichment of B chromosomes in chromosome-specific repetitive sequences is a striking and conserved feature that has been observed across taxa (**Figure 2D**). In *Nasonia vitripennis*, PSR is dominated by complex satellite repeats (PSR2, PSR18, PSR22), which have been proposed to act as molecular sinks for chromatin-associated factors, thereby contributing to genome elimination (Eickbush et al., 1992; Nur et al., 1988; Dalla Benetta et al., 2020). Similarly, rye B chromosomes harbour satellite families such as D1100 and E3900 within the distal control region, while maize and sorghum B chromosomes contain specific (peri)centromeric repeats (e.g. ZmBs in maize and SpuCL166 in wild sorghum) (Blavet et al., 2021; Chen et al., 2024; Bojdová et al., 2026). In *Drosophila melanogaster*, genomic analyses revealed that the B chromosome likely originated from chromosome 4 and is largely composed of chromosome 4-derived repetitive and heterochromatic sequences (Hanlon et al., 2018), further supporting the notion that repeat accumulation is a common feature of B chromosome evolution.

These observations raise the possibility that repetitive DNA provides a molecular basis for chromosome recognition by the cellular machinery as a prerequisite for any chromosome-specific behaviour. However, the mechanisms by which B chromosomes are selectively identified and targeted remain unresolved.

## 6 Conclusion

B chromosomes, along with other non-canonical chromosomal elements, demonstrate that chromosome transmission is not solely dictated by Mendelian laws but can be actively manipulated through a diverse set of molecular strategies. Based on current knowledge, it is almost certain that various B chromosomes have developed different molecular tools during evolution affecting their own inheritance. Far from being inert passengers, these elements exploit chromatin plasticity, spindle dynamics and cell cycle states to bias their own inheritance. The evidence reviewed here shows that chromosome-specific behaviour can be mediated by multiple layers of regulation and occur likely through interacting pathways and mechanisms. However, despite increasing knowledge, we still have uncovered only a few pieces of this puzzle and there is a long way to gather all the evidence and satisfactorily describe the whole mechanisms. Trends in the B chromosome biology aims towards the functional validation of candidate genes implicated in chromosome drive and elimination, particularly those associated with centromere function, cohesion dynamics and chromatin regulation. Another important challenge will be to determine how these molecular pathways interact to generate chromosome-specific behaviours at the cellular level. Finally, comparative genomic and epigenomic analyses across independently evolved B chromosome systems will be essential to distinguish lineage-specific adaptations from conserved molecular principles underlying chromosome drive and elimination.
